# Bio-Inspired Micro-Fluidic Angular-Rate Sensor for Vestibular Prostheses

**DOI:** 10.3390/s140713173

**Published:** 2014-07-22

**Authors:** Charalambos M. Andreou, Yiannis Pahitas, Julius Georgiou

**Affiliations:** Department of Electrical and Computer Engineering, University of Cyprus, Nicosia 1678, Cyprus; E-Mails: pahitas@ucy.ac.cy (Y.P.); julio@ucy.ac.cy (J.G.)

**Keywords:** vestibular prostheses, oscillopsia, inner-ear semi-circular canals, angular-rate sensor, implantable bio-sensors, gyroscope, inertial sensor, motion sensor, MEMS, microfluidics

## Abstract

This paper presents an alternative approach for angular-rate sensing based on the way that the natural vestibular semicircular canals operate, whereby the inertial mass of a fluid is used to deform a sensing structure upon rotation. The presented gyro has been fabricated in a commercially available MEMS process, which allows for microfluidic channels to be implemented in etched glass layers, which sandwich a bulk-micromachined silicon substrate, containing the sensing structures. Measured results obtained from a proof-of-concept device indicate an angular rate sensitivity of less than 1 °/s, which is similar to that of the natural vestibular system. By avoiding the use of a continually-excited vibrating mass, as is practiced in today's state-of-the-art gyroscopes, an ultra-low power consumption of 300 μW is obtained, thus making it suitable for implantation.

## 1. Introduction

**Figure 1.**
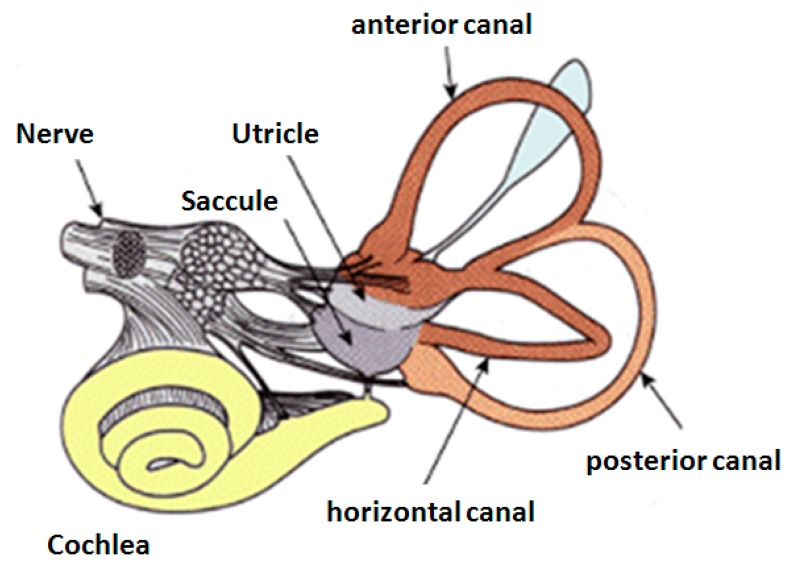
Illustration of the human vestibular system and its components [10].

People suffering from oscillopsia, vertigo and loss of balance due to a vestibular-related disorder could benefit from the development of a vestibular neural prosthesis. The inner ear's vestibular system provides cues about self-motion and balance, body posture and helps stabilize vision during movement. Damage to this system causes failure of vestibulo-ocular and vestibulo-spinal reflexes which results in dizziness, vertigo, nausea, imbalance, blurred vision and instability in locomotion [1]. The restoration of vestibular system can therefore be achieved by bypassing a dysfunctional element in the vestibular pathway and using artificial stimulation [1–7]. There are a number of sites along the vestibular pathway that can be tapped into, like the ampullae, the scarpa's ganglion and individual nerve bundles. A detailed anatomy of the human vestibular system is illustrated in Figure 1. A key component that is required to enable the creation of an ergonomic vestibular prosthesis is that of a suitable Micro-Electro-Mechanical-System (MEMS)-based angular-rate sensor or gyroscope [8,9], since existing gyroscopes are too power-hungry for a totally implantable solution.

Low-power, low-cost, small-footprint gyro development has been predominantly driven by the mobile phone and gaming industry. However the power and reliability requirements, in these industries, are far less stringent than those required for implantable devices. In vestibular prototypes, the gyro tends to consume most of the supplied power [11], and as such, the lack of a suitable off-the-shelf gyro is considered one of the stumbling blocks in the development of a fully implantable vestibular prosthesis. State-of-the-art gyros avoid utilizing large masses by vibrating small masses and exploiting the Coriolis effect [12–18]. Vibrating structures need voltages as high as 40V to electro-statically stimulate the vibration [19]. In addition, vibrating these structures at hundreds of kilohertz, for over a decade, is likely to cause fatigue and premature failure of the sensor [20,21].

Although non-vibratory structures have been explored, so far no proof-of-concept gyroscopic device has given promising results [22–25]. Thermal based flow sensing [24,25] is too power hungry and has not been shown to provide the required sensitivity for this application. Various artificial haircell flow sensors [26], have been constructed by growing “cilia” perpendicular to the silicon wafer plane. Using such an approach for sensing motion in a gyroscopic device increases the complexity and cost of fabrication. Furthermore some of these “cilia” that are built with SU-8 epoxy [27] are very likely to detach from the silicon sensing element over time, especially if immersed in a fluid. The surface acoustic wave (SAW) based device, behaves much like a vibrating structure, in that it needs energy to continually keep the atoms in motion via high frequency surface acoustic waves (160 MHz) [28].

In this paper we present a simple but elegant MEMS-Microfluidic solution, which consumes significantly less power than vibration based gyroscopes. The initial concept of this work was previously presented in 2013 [29], with only the simulated results available at that time. The proposed solution mimics the natural mechanism of the vestibular system where a sealed micro-channel is filled with a liquid. Any rotation of the system about the axis of the channel results in a relative motion between the channel itself and the liquid, due to the fluid's inherent inertia. A flexible object fixed to the channel walls can sense the fluid flow upon deflection, e.g., with a piezo-resistor.

## 2. Proposed Bio-Inspired Angular-Rate Sensor

The proposed micro-fluidic gyro is based on a relatively large fluidic mass that deflects a sensing element similar in principle to a biological semicircular canal (SCC). The fluid dynamics of a SCC can be mathematically expressed with the “torsion pendulum equation” [30] as:

(1) $$I\ddot{\theta }+B\dot{\theta }+K\theta =-I\alpha$$

where θ is the mean angular displacement, $\dot{\theta }$ is the mean angular velocity and is $\ddot{\theta }$ the mean angular acceleration of the endolymph fluid. *α* is the angular acceleration of the head perpendicular to the plane of the canal, *I* is the inertia, B is the damping coefficient and *K* is the stiffness of the cupula (in the case of the proposed gyroscope, it's the effective stiffness of the fluid-intercepting cantilevers).

Taking this phenomenon into account, the proposed design integrates flow sensors in the path of the liquid inside the micro-channel. These sensors are cantilevers with embedded piezo-resistors, whose resistance changes according to the deformation caused by pressure applied across the sensing structure.

**Figure 2.**
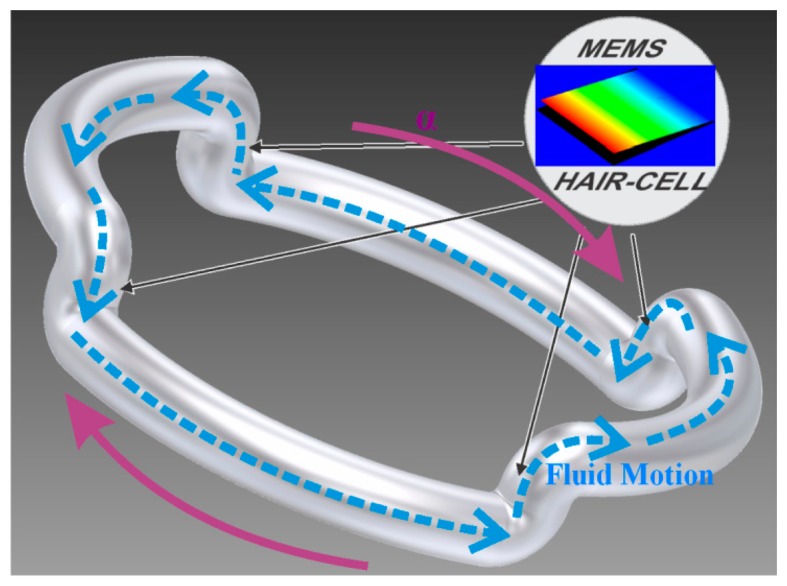
Illustration of the principle behind the sensor, which integrates four cantilevers (MEMS hair cells) at each of the layer transition points, *i.e.*, in the bulk-micromachined silicon layer, positioned at the locations shown by the black arrows.

**Figure 3.**
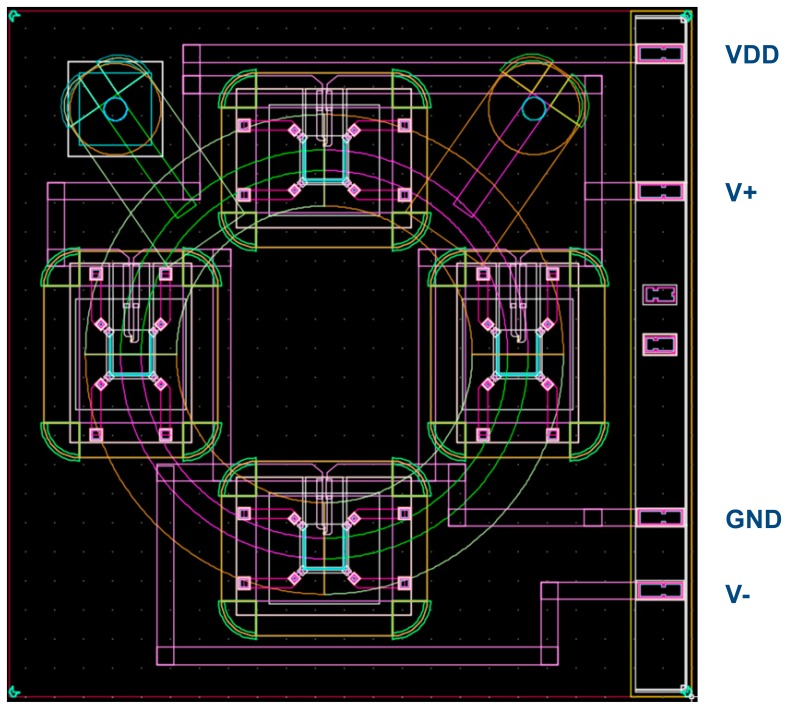
Actual physical layout of the structure consisting of: (1) top-glass canal (green quadrants in upper-left and bottom-right), (2) bottom-glass canal (lilac quadrants in bottom-left and upper right), (3) bulk micromachined silicon layer connecting upper and lower quadrants, intercepted by four flaps (light blue Π-shaped cuts in a 3.1 μm silicon membrane) that are oriented in same direction in all cases and which contain a piezo-resistor centered on each of the membrane's uncut side, (4) various conductors for powering the Wheatstone Bridge (VDD, GND) and differential outputs (V+, V−).

The hybrid MEMS-microfluidic structure is illustrated in Figure 2 while the physical layout of the design is shown in Figure 3. This structure uses a fluidic mass that is contained in etched glass layers, which are anodically bonded on top and below a bulk-micromachined silicon layer, and whose deflectable structures include buried piezo-resistors for sensing. The deflectable structures consist of four cantilevers (flaps) that intercept the path of the fluid as it flows, serving as the cupula and one piezo-resistor in each cantilever, serving as the hair cells of the SCC.

When angular acceleration is present, the liquid in the quarter-torroidal cavity in the top glass, is deflected through the middle silicon bulk-micromachined layer, where it will deflect a cantilever containing a buried piezo-resistor and progress through the bottom quarter-torroidal cavity etched in the bottom glass, after which it will be deflected back to the top glass cavity, again via the bulk-micromachined layer containing cantilevers and so on. Thus, liquid completes a loop through a distorted torroid, passing through the silicon layer that contains the sensing structures (equivalent to cupula and hair cells).

Angular rotation of the whole device creates an inertial force, which increases the pressure on the sensing cantilever, leading to a detectable deflection that is proportional to the angular acceleration. To cope with the resistors' process variations, the strain gauges are designed in pairs, to form a Wheatstone bridge, which converts the strain induced resistance variations into output voltage variations. A differential voltage read-out circuit then reduces the effects of semiconductor process variations. The sensing piezo-resistive strain gauges are strategically located at regions of maximum stress “σ”, which are at the perimeter edges of the cantilever, to give increased electrical sensitivity.

In the sensor of Figures 2 and 3, all four piezo-resistors are placed longitudinally to the stress axis, oriented parallel to the 110 crystallographic direction, in order to maximize the coefficient of piezo-resistivity. During angular motion, two piezo-resistors undergo tensile stress (top to bottom flow), whilst the other two undergo compressive stress (bottom to top flow). These are wired internally, via buried conductors, so as to form a Wheatstone bridge. Thus the two resistive divider legs will determine the output voltage as:

(2) $$\Delta V_{\text{out}}\propto \Delta R\propto \Pi_{L}\sigma_{L}+\Pi_{\tau }\sigma_{\tau }\propto \Pi \left(P-P_{0}\right)$$

where Π is the piezo-resistive coefficient, σ is the mechanical stress, subscripts L and τ denote the longitudinal and transversal coefficients respectively, along the resistor axes, and (P − P_0_) is the differential pressure on the sensing element. The thickness of the silicon cantilever (hair-cell) is 3.1 ± 0.3 μm.

**Figure 4.**
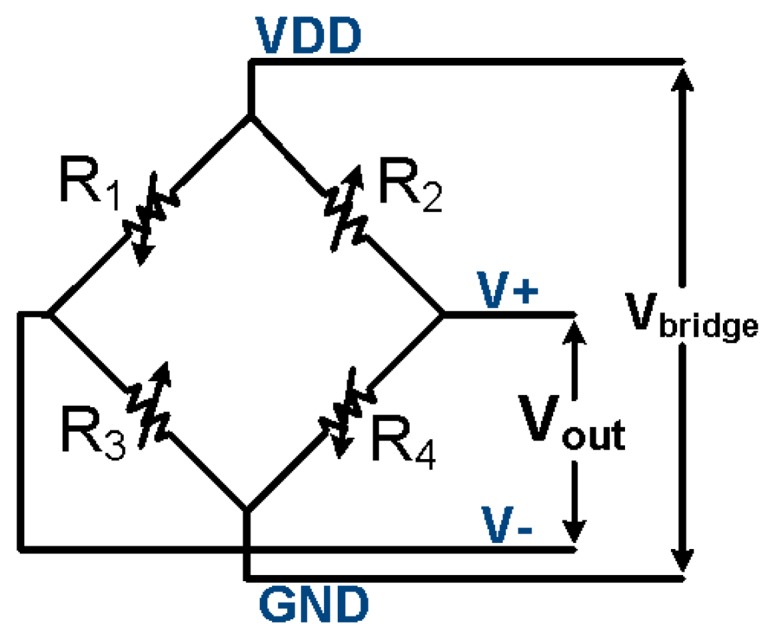
Wheatstone bridge.

The output voltage of the Wheatstone bridge of Figure 4 that was used in the proposed structures of Figures 2 and 3 is described by:

(3) $$V_{\text{out}}=V_{\text{bridge}}\left(\frac{R_{3}}{R_{1}+R_{3}}-\frac{R_{4}}{R_{2}+R_{4}}\right)$$

The proposed approach allows an increased inertial (fluidic) mass and thus provides the required sensitivity without utilizing vibrating structures and the Coriolis effect. A key concept of this solution is the decoupling of the structure that holds the (large) mass from the sensing structure; the fluid is supported by the walls of the etched glass, which at the thinnest point is about 200 μm thick, thus providing enough support to the large mass, so as avoid damage if the device is dropped onto a hard surface. The sensing structure thickness is only a few micrometers, but as it is not firmly attached to the mass, it is not susceptible to breakage when dropped.

## 3. Testing Procedure

Given that the MEMS-microfluidic angular rate sensor was a completely novel design, it follows that some challenges were expected to appear whilst attempting to test the functionality of the device.

The working principal of the device lies in the premise that the circular channel is filled with a liquid. For the purposes of these first tests, aimed at a proof-of-concept device, deionized water was chosen for its physical properties—density and viscosity, its chemical proximity to the endolymph and its bio-compatibility/non-toxicity.

### 3.1. Filling the Structures

**Figure 5.**
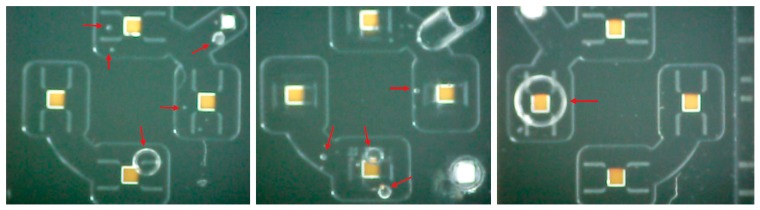
Micro-bubbles that can appear with incorrect filling methods, in the MEMS-microfluidic sensors. The micro-bubbles are indicated with red arrows (photographs).

The proposed structure has two holes that lead from the top external surface to the circular channel. Theoretically, during the filling process this allows for the liquid to enter from one hole, while air is pushed out from the other. Due to the microscopic level of the structure, dislodging the air micro-bubbles was not straight forward. Air micro-bubbles are held in place by forces of adhesion between the liquid around the bubbles and the channel structure as shown in Figure 5. In addition, the cantilevers presented an obstacle to the flow of water and hence the bubbles.

After testing various filling methods, it was found that the most effective method for filling the micro-fluidic channels, without any trapped micro-bubbles, required the use of a vacuum chamber. This approach for filling the device involves immersing the device in deionized water and placing it in a vacuum chamber. Air is then pumped out of the chamber thereby causing the trapped air in the device to expand and escape. Upon re-pressurization to normal atmospheric pressure, the remaining air is compressed and dissolved in the water drawn into the device. The level of vacuum used was carefully chosen based on the temperature of the chamber to avoid boiling off the water at the lower pressure. The rate of re-pressurization is an important parameter in this process, since if the flow of water is too fast it can damage the MEMS sensing structures. By using this method, the MEMS device was completely filled with deionized water. Furthermore it is fast, easy to set-up and can be scaled up to fill multiple devices simultaneously in a mass production scenario.

### 3.2. Sealing the Structures and Assembling the Test Board

In order to characterize the sensor it has to be ensured that the device remains full with water, without any micro-bubbles for the duration of few weeks. The seal applied for testing purposes was an epoxy resin based UV-A cured adhesive—Panacol Vitralit 1657. Using this method, air only first appeared within the channel a few months after the sensor was sealed. A permanent hermetic seal involves adding an additional glass layer over the holes and using a UV laser to fuse the glasses together, without significantly heating the fluid.

**Figure 6.**
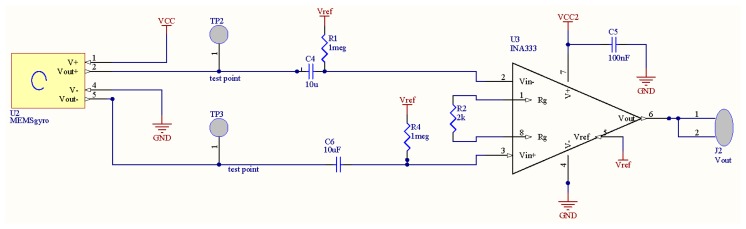
Schematic design of the PCB test board for testing the proposed sensor.

The printed circuit board (PCB) as shown in Figure 6 is designed such that the MEMS device is electrically connected to the PCB through the process of wire bonding. The applied methodology first requires that the die is secured to the PCB with die-attach epoxy (CW200). Next the die is wire-bonded to the board and the wire-bonds are protected with Panacol Vitralit 1671 epoxy. The sensor is subsequently subjected to the above proposed filling and sealing processes.

## 4. Simulated and Measured Results

**Figure 7.**
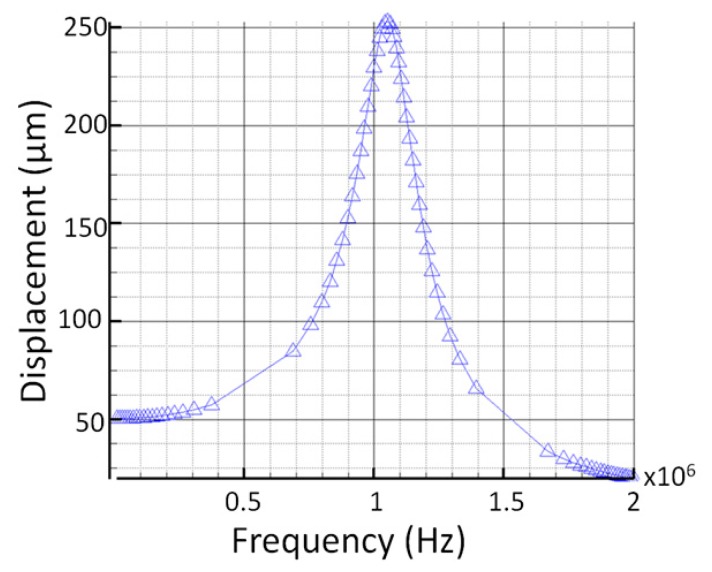
Simulated frequency response of the sensing elements of the proposed sensor.

The simulated frequency response of the sensing elements (cantilevers) of the angular rate sensor, after being designed, was simulated using the Coventor MEMS design software. The displacement *versus* frequency is shown in Figure 7, where the resonant frequency is at 1.05 MHz, well above the frequencies of interest.

**Figure 8.**
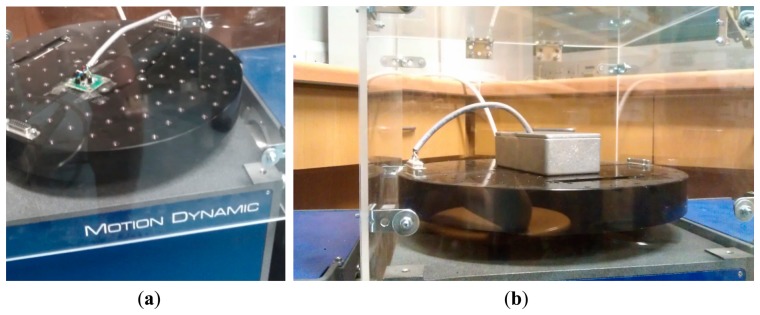
Test setup for characterization of the proposed angular rate sensor. (**a**) the PCB with the sensor is mounted on top of the rate-table; (**b**) the PCB with the sensor is placed in an aluminum box and mounted on top of the rate-table. The aluminum box ensures electromagnetic shielding of the device under test so as to avoid picking up electrical noise emitted from the rate-table motor.

**Figure 9.**
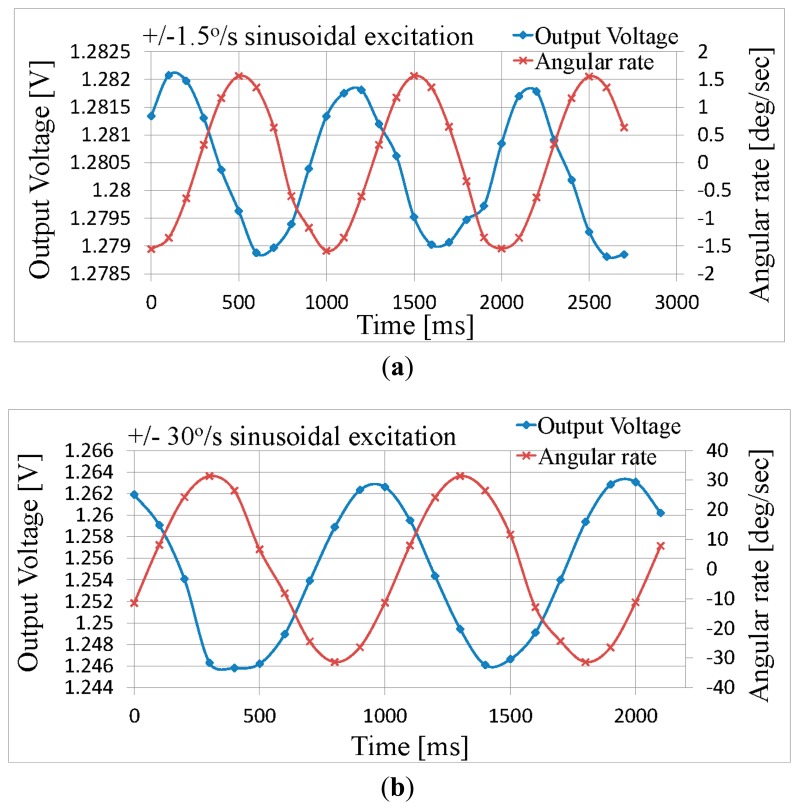
Measured results of the output voltage *vs.* angular rate. (**a**) Angular rates between 0 °/s and a maximum of ±1.5 °/s (sinusoidally modulated); and (**b**) Angular rates up to ±30 °/s (sinusoidally modulated).

The angular-rate sensitivity of the sensor can be modified through the selection of the fluidic mass, depending on its density and viscosity. The proposed angular rate sensor was tested after being filled with deionized water and sealed. A PCB integrating the proposed sensor and an instrumentation amplifier was prepared for the measurements. The system was tested using a Tes-3T Motion Dynamic rate table, as shown in Figure 8. The sensor's Wheatstone bridge output was amplified by an instrumentation amplifier, and routed to the inputs of a National Instruments acquisition card, via the wiring and slip-rings built into the rate table. The motion and the corresponding measurements were controlled through customized Labview scripts that ensured accurate synchronization of the motion and the acquired measurements. The measured results are illustrated in Figure 9a,b, which demonstrate that the sensitivity of the system is comparable to that of humans, whilst keeping the total power consumption down to 300 μW @ 2V, per axis, including the instrumentation amplifier. The power consumption can be attributed to that consumed by the continuous Wheatstone Bridge bias and that of the instrumentation amplifier; in the current proof-of-concept prototype the instrumentation amplifier consumes 100 μW @ 2V, whilst the continuous Wheatstone Bridge bias consumes 200 μW @ 2V. To reduce the power further one could power cycle the Wheatstone Bridge bias to match the sampling rate of the A/D converter. The exact duty cycle would depend on the maximum integrated piezo-resistor value and the maximum capacitance associated with the output node. Furthermore it is possible to reduce the power supply voltage further at the expense of the device sensitivity (see Equation (3)).

Inherent resistor mismatch between the two legs of the Wheatstone bridge create a small DC component at the output of the sensor. Thus it is necessary to either feed the signal into a high-pass filter (cut-off frequency of 0.1 Hz) prior to being amplified or use an AC coupled amplifier to measure the output of the microfluidic angular rate sensor.

Figure 9a shows the analog output *versus* time when the sensor is subjected to an angular rate between 0 °/s and a maximum of ±1.5 °/s (sinusoidally modulated). It is clear that the target sensitivity of 1 °/s, which is approximately that of humans, is easily achievable, even without any signal conditioning (e.g., averaging, filtering *etc.*). Similarly, Figure 9b shows the response of the sensor which is subjected to an angular rate reaching up to ±30 °/s (sinusoidally modulated). For the angular rate of ±1.5 °/s an output voltage of ±1.5 mV is measured, while for ±30 °/s an output voltage of ±8 mV is measured. The approximate 90° lead of the output voltage with respect to the angular rate is because this sensor responds to angular acceleration as described in Equation (1); the angular acceleration is the derivative of the angular rate *i.e.*, the derivative of sinusoidal angular rate input appears as a cosine output voltage. Due to the mechanism used to sense the angular rate the output is not expected to be linear, however this is not a problem for vestibular implants, since the natural system behaves in a similar manner *i.e.*, a form of dynamic range compression takes place. If required, the analog output can be digitalized with a slight increase of the power budget, e.g., 3.1 μW if use MAX1393 ADC at 1.8V with sampling rate of 1 Ksps, totaling approximately 0.3 mW per axis. A 3-axis angular-rate sensor could be realized by mounting three of the proposed sensors perpendicular to each other, within the vestibular implant package, much like the physiological vestibular organ. Alternatively, monolithical integration of these gyros for 3-axis sensing is also possible using the same process, however the sensitivity of other axes would be compromised since the torroidal shape would be greatly deformed and at most two “haircells” could be used.

**Figure 10.**
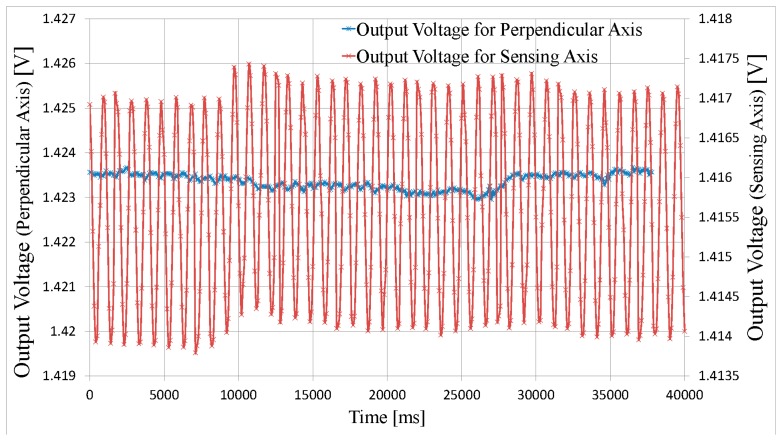
Measured sensor output voltage for angular motion stimuli on the sensing axis and for angular motion stimuli on the cross-axis (the sensor is subjected to the same modulated angular rate of ±3 °/s for both, in-plane and perpendicular motion).

The natural vestibular system detects angular rates in three perpendicular axes, whilst demonstrating low cross-axis sensitivity. Thus it is important that any rotation that is perpendicular to the sensing axis, of the bioinspired gyroscope presented, should not be detected, *i.e.*, the sensor should have a very low cross-axis sensitivity. In order to verify this feature, the test assembly was designed so as to be able to also mount it perpendicular to the plane of motion of the rate table, and thus be able to test cross axis sensitivity. The measured results of this test are shown in Figure 10, where the sensor was subjected to an amplitude modulated angular rate reaching up to ±3 °/s, for both the sensing axis and a perpendicular axis. It is clearly seen that the cross-axis sensitivity is quite low.

**Figure 11.**
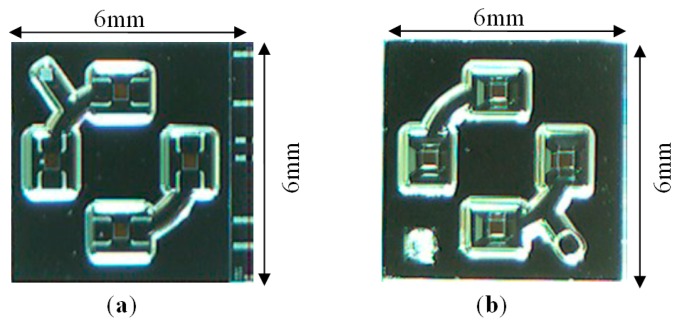
Microphotographs of the gyroscopic sensor structure. (**a**) Top view of the gyro; (**b**) Bottom view of the gyro.

The above tests and measurements demonstrate that this bioinspired, proof-of-concept vestibular gyroscope, fabricated with Sensonor's standard MEMS technology, has much potential and functions very similarly to the predictions made with back-of-the-envelope calculations [29]. The proposed angular rate sensor has also demonstrated that it can clearly detect motion, even at angular rates below 1 °/s without needing to actively excite the sensing mass, thus providing significant power savings. The MEMS technology used to fabricate the sensor utilizes doped p-type conductors within an n-type substrate for signal routing. The result is that several reverse biased parasitic diodes exist in the structure, which is noisier than if metal routing was used. We believe that with a customized MEMS process the performance of this gyroscope could be improved further, allowing the size to be reduced. Furthermore the measured analog signal was obtained through a relatively noisy connection, provided via the slip rings of the rate table. A summary of the sensor's specifications is given in Table 1. The total area of the sensor die is 6 mm × 6 mm, as shown in Figure 11a,b. This is approximately the same size as the natural semicircular canals. Similarly the sensor sensitivity is approximately that of the natural vestibular system *i.e.*, 1–2 °/s [31].

## 5. Conclusions

A bio-inspired, proof-of-concept, ultra-low power, MEMS-microfluidic angular-rate sensor, which does not require continually vibrating structures, has been designed, simulated, fabricated and successfully tested. The size of the chip is 6 mm × 6 mm including the pads area and it consumes a mere of 300 μW at 2 V. The proposed gyroscope's low-power consumption and comparable performance to the vestibular system, which is able to detect angular rates as low as 1 °/s, may enable totally implantable vestibular implants to be developed in the near future. This principle may also be applied to other applications where stringent power constraints prohibit the use of existing gyroscope technology.

## Figures and Tables

**Table 1.** Summary of the Specifications of the proposed angular-rate sensor.

**Parameter**	**Performance**
Sensitivity	< 1 °/s
Supply Voltage	2V
Power Consumption	300 μW
Cantilever Thickness	3.1 ± 0.3 μm
Chip Size (Area)	6 mm × 6 mm
Technology	Sensonor's standard MEMS
